# *iTools*: A Framework for Classification, Categorization and Integration of Computational Biology Resources

**DOI:** 10.1371/journal.pone.0002265

**Published:** 2008-05-28

**Authors:** Ivo D. Dinov, Daniel Rubin, William Lorensen, Jonathan Dugan, Jeff Ma, Shawn Murphy, Beth Kirschner, William Bug, Michael Sherman, Aris Floratos, David Kennedy, H. V. Jagadish, Jeanette Schmidt, Brian Athey, Andrea Califano, Mark Musen, Russ Altman, Ron Kikinis, Isaac Kohane, Scott Delp, D. Stott Parker, Arthur W. Toga

**Affiliations:** 1 Center for Computational Biology, University of California Los Angeles, Los Angeles, California, United States of America; 2 National Center for Integrative Biomedical Informatics, University of Michigan, Ann Arbor, Michigan, United States of America; 3 National Center for Multi-Scale Study of Cellular Networks, Columbia University, New York, New York, United States of America; 4 National Center for Biomedical Ontology, Stanford University, Stanford, California, United States of America; 5 Center for Physics-based Simulation of Biological Structures, Stanford University, Stanford, California, United States of America; 6 National Alliance for Medical Imaging Computing, Harvard University, Cambridge, Massachusetts, United States of America; 7 Informatics for Integrating Biology and the Bedside, Harvard University, Cambridge, Massachusetts, United States of America; 8 Neuroscience Center, Massachusetts General Hospital, Boston, Massachusetts, United States of America; 9 National Center for Microscopy Imaging Research, University of California San Diego, San Diego, California, United States of America; Genome Institute of Singapore, Singapore

## Abstract

The advancement of the computational biology field hinges on progress in three fundamental directions – the development of new *computational algorithms*, the availability of informatics resource *management infrastructures* and the capability of *tools to interoperate and synergize*. There is an explosion in algorithms and tools for computational biology, which makes it difficult for biologists to find, compare and integrate such resources. We describe a new infrastructure, *iTools*, for managing the query, traversal and comparison of diverse computational biology resources. Specifically, *iTools* stores information about three types of resources–data, software tools and web-services. The *iTools* design, implementation and resource meta - data content reflect the broad research, computational, applied and scientific expertise available at the seven National Centers for Biomedical Computing. *iTools* provides a system for classification, categorization and integration of different computational biology resources across space-and-time scales, biomedical problems, computational infrastructures and mathematical foundations. A large number of resources are already *iTools*-accessible to the community and this infrastructure is rapidly growing. *iTools* includes human and machine interfaces to its resource meta-data repository. Investigators or computer programs may utilize these interfaces to search, compare, expand, revise and mine meta-data descriptions of existent computational biology resources. We propose two ways to browse and display the *iTools* dynamic collection of resources. The first one is based on an ontology of computational biology resources, and the second one is derived from hyperbolic projections of manifolds or complex structures onto planar discs. *iTools* is an open source project both in terms of the source code development as well as its meta-data content. *iTools* employs a decentralized, portable, scalable and lightweight framework for long-term resource management. We demonstrate several applications of *iTools* as a framework for integrated bioinformatics. *iTools* and the complete details about its specifications, usage and interfaces are available at the *iTools* web page http://*iTools*.ccb.ucla.edu.

## Introduction

Bioinformatics is quickly becoming the central *core* that integrates the many disparate bodies of data, scientific knowledge and computational infrastructure from fields as diverse as genetics, structural biology, medical and animal models of disease, imaging, engineering, etc. For example, biological imaging and shape representation [1]–[3], sequence analysis [4], regulatory genomics [5] and alternative splicing [6] are critical components of computational biology. Many space, time, function or interaction modeling techniques apply across the vast spectrum of scales from genotypes to phenotypes, from the small scale of microarray imaging for genomics, to the larger *in vivo* neuroimaging scale.

In this manuscript, we discuss challenges of development, maintenance and dissemination of robust computational biology resources (data, tools and services, see below). The rapid growth, usage specifications, application domain and method diversity of contemporary computational biology resources presents an impediment to finding, comparing and integrating such data and tools. We propose a new decentralized, extensible, lightweight, scalable and portable framework (*iTools*) to enable resource location, management, evaluation and mediation. *iTools* aims to provide an open, yet secure, infrastructure for resource archival, retrieval and traversal; communication between tool developers, clinicians, scientists and the general community; and to facilitate the interoperability of resources developed by a wide spectrum of investigators and employed in a variety of computational biology studies. There is an increasing need in the bioinformatics community for accurate accounting of data and resources, e.g., the bioinformatics Resourceome effort [7].

We leveraged the broad research, computational, applied and scientific expertise available at the seven National Centers for Biomedical Computing (NCBC) to design, implement and validate a new schema for classifying, categorizing and integrating different computational biology resources across space-and-time scales, biomedical problems, computational infrastructures and mathematical foundations. The proposed infrastructure is not limited to needs and applications of the NCBCs. Rather it is intentionally designed to be extensible and applicable to broader range of biomedical research.

### National Centers for Biomedical Computing (NCBCs)

Seven NCBCs were funded by NIH as part of the Roadmap for Medical research in 2004 and 2005. The computational biology research at these centers and their partner and collaborating institutions spans a wide range, which ensures that the NCBC program provides a foundation for a National infrastructure for diverse biomedical computing. The research and clinical goals, project descriptions, home-pages and the computational infrastructures provided by of all NCBC centers is available online at http://www.NCBCs.org.

### Current approaches for software tool classification, categorization and integration

There are a number of efforts to develop integrated resources that provide a common user-friendly access to variety of tools and software programs for computational biology. **Table 1** summarizes many of these attempts. These are important efforts to catalog computational tools; however, there are remaining challenges, whose solutions may significantly affect both the development of new tools and technologies and the utilization of existent novel computational biology resources. For example, there is no one-stop site for researchers to go to search, contrast and integrate tools. Many sites specialize on certain types of tools, and researchers must know about these sites to find the tools they seek. In addition, graphical traversal, semantic search, and effective interfaces for tool integration are still mostly envisioned for the future. Research progress also may be significantly potentiated by providing agile interfaces, extensibility and portability features to any new meta-resource of computational biology tools and software.

**Table 1:** Examples of Meta-Resources for Computational Biology.

Name & URL	Community Built	Open Source Devel	Enables Resource Comparison	Machine Interfaces	Fields
*iTools*, *iTools*.ccb.ucla.edu	Y	Y	Y	SOAP/WSDL/HTML/XML	General
IATR, www.cma.mgh.harvard.edu/iatr	Y	N	N	HTML/XML	Neuroimaging software
SfN NDG Software Database, ndg.sfn.org/	Y	N	N	N	Neuroscience tools & data
BIAS, www.mcb.mcgill.ca/bias/	Y	Y	N	N	Bioinformatics
NIF, neurogateway.org/	Y	N	N	HTML/XML	Neuroscience
NCI GForge, gforge.nci.nih.gov/	Y	Y	N	HTML	Computational Biology
Source-Forge, www.sourceforge.net/	Y	N	N	HTML	General
caBIG Resources, cabig.nci.nih.gov/	Y	Y	Y	HTML	Bioinformatics tools & data
NCICB Resources, ncicb.nci.nih.gov/NCICB/tools	Y	N	N	HTML	Translational tools
Guide to Available Math Software (GAMS), gams.nist.gov/HotGAMS	N	N	N	HTML/Java	Mathematics
Indiana University Biology Software archive, iubio.bio.indiana.edu/software/	Y	N	N	FTP/HTML	Biology & chemistry
NeuroInformatics Pilot Project, www.neuroinf.de/software/topic_view	Y	Y	N	N	General
SimTK, http://simtk.org	Y	Y	N	HTML	Physics simulation

Summary comparing *iTools* to other similar meta-resources environments for archival and retrieval of software tools for computational biology.

### Goals of this research

Our overarching aim is to build a meta-resource that catalogs computational biology data, tools and services provided by the computational biology community. More specifically, our goal is to implement *iTools* as an extensible meta-resource, validate it using NCBC resources, and enable its growth in depth and breadth in the future. Additional goals of the *iTools* resource are to:

Develop a general, scalable and decentralized *ontology* of computational biology resources (NCBC Biomedical Resource Ontology http://Bioportal.Bioontology.org), which can be used in the context of a flexible XML resource description and hastens the creation of plug-ins and an interface infrastructure for utilization by an external computer program.

Develop a *database* of XML descriptions of computational biology resources (adhering to an XML data type) general enough to describe most types of existing resources. This database should allow an easy and robust access (manual or computer-initiated) to the resource descriptions, include powerful and user-friendly search and download interface, provide an agile syntax of the XML descriptors, and facilitate the meta-resource curation, community editing and evaluation. The latest version (V4) of this XML is located at the NCBC biositemap site www.biositemap.org.

Develop the infrastructure for integration and interoperability of meta-algorithms for biomedical computing. This is a high-level goal that aims to facilitate the communication between different tools in terms of meta-data resource descriptions (e.g., software tool I/O types) useful for streaming data from one resource to the next (perhaps facilitated by data mediation modules).

## Methods

To address our goals, we designed and implemented an infrastructure, *iTools*, which is extensible, portable and scalable, and directly addresses these challenges. Below we describe the specifications, versions, features, functionality and utilization of *iTools*.

### Types of Computational Biology Resources

At the highest level, we distinguish three types of computational biology resources –* data*, *tools* and *services*.

(Downloadable) *Data Resources* type includes observed biomedical (raw) data, which is typically fed as input in different computational tools; model data, processed data resulting as an output from various tools (e.g., atlases); and textual data, spread sheets, web-pages (e.g., clinical charts). Computational biology data may be saved in any of a large number of formats, data types and structures. For example, XML (eXtensible Mark-up Language), JavaScript Object Notation (JSON) objects, binary or ASCII data, or network-based serialization formats such as Simple Object Access Protocol (SOAP), Web Service Definition Language (WSDL), BioMOBY [8], etc.

*Tools* (software resources) consist of computational algorithms (e.g., Level-sets, [9]), software suites (e.g., geWorkbench [10]), downloadable libraries (e.g., ITK, [11]) and locally executable programs (e.g., Debabeler, [12]).

*Web Services* consist of algorithms and applications that can perform remotely, communicate using web protocols, are built on top of existing XML standards, and allow interactions of incompatible applications. Web services simplify this communication by relying on a standardized mechanism for description, location and communication of different online applications. Common web services frameworks consist of three components – communication protocol, service description and service archival/traversal [13].

### *iTools* Specifications

*iTools* is an infrastructure that manages the description of computational biology resources (data, tools, and services). *iTools* has a (human) graphical user interface and provides additional (machine) interfaces to the underlying database for browsing, revisions and updating by external computer programs. *iTools* includes XML, SOAP and WSDL machine interfaces to its database. There are three *types of iTools users* – expert editors, registered and general users. Only editors may update the *iTools* resource descriptions. Registered users may submit new resource descriptions (or updates), retrieve or comment on resources and their descriptions. General users may only browse the *iTools* database (via any of the provided human or computer interfaces). New submissions/revisions are submitted, reviewed and handled appropriately by the expert editors. Most users will require the *iTools* Java web interface to search, explore and review computational biology resources. Machine utilization of *iTools* is expected to become more prevalent as the *iTools* usage increases. *iTools* is intended to be a community generated meta-resource. We simply built its infrastructure; the user community will populate and manage this environment and its content. *iTools* collects, manages and disseminates meta-data about diverse computational biology resources, which makes *iTools*, itself, a dynamic biomedical resource. The complete *iTools* specifications and UML design are available from the *iTools* web-page (http://cms.loni.ucla.edu/iTools/Specs.aspx) and may be used to discover *iTools* functionalities and develop new plug-in interfaces.

### *iTools* Functionalities

As a meta computational biology resource, *iTools* is completely dependent on the volume, quality and quantity of the computational tools included in it. For this reason we have designed *iTools* to allow flexible and dynamic access to/from its underlying database. The *iTools* configuration file allows one to specify one of several background or file-system database choices. In addition, we allow several types of human and computer interfaces to the *iTools* database. All of these are publicly and anonymously accessible, except for the *iTools update* functionality, which requires expert user authentication.

**Figure 1:**
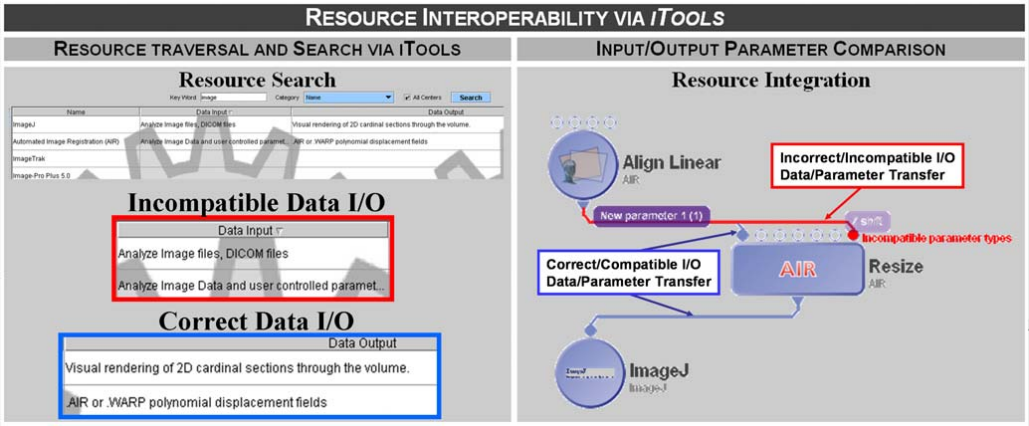
Left *panel* shows the search, traversal and comparison of tools (in this case image alignment and visualization) based on their data input/output specifications. The *right* panel illustrates how streaming data through independent tools (via an external graphical workflow environment, e.g., LONI Pipeline) may be facilitated by the types of data I/O parameters stored as iTools resource-specific meta-data.

The key *iTools* functionalities are **storage, searching, traversal and retrieval** of resources and their corresponding meta-data description. *Storage* refers to the *iTools* repository function which allows entering new and updating existent resources and resource-descriptions (resource meta-data). *Searching* is the major end-user functionality which allows quick and efficient finding of appropriate computational biology resources by keywords, description, authors, versions and many other of the resource meta-data fields. *Traversal* is a function that allows exploration of resources, comparison of similar resources and investigation of relation and interoperability of pairs of resources within *iTools*. Traversal is facilitated via the NCBC Ontology hierarchy, the ordering of the resources in the tabular-view according to any of the user-selected meta-descriptions or via the Interactive Hypergraph *iTools* viewer. The search functionality enables inquiry for resources based on specific textual input (e.g., keyword, author, etc.) Traversal is a graphical exploration of the available resources, using organization-centric or ontology-based hyperbolic graph view, or via tabular view (regular resource-view tab). **Figure 1** shows how resource-interoperabilities may be explored within the *iTools* regular resource-view tab, by comparing resource input and output data specifications. The *iTools* ability to mediate resource comparison and integration is dependent on the volume and complexity of the resource's meta-data, e.g., number, type, specification and requirements of inputs and outputs.

### *iTools* Resource Updates

*iTools* provides a number of mechanisms for updating the resource meta-data descriptions, stored in the *AllResources* and *AllProperties* database fields (http://cms.loni.ucla.edu/iToolsDatabase.aspx). The most common interfaces for adding new and updating existing resources are manual user input and the automatic computer updates. The former is initialized by double-clicking on any resource (a leaf-node, not a directory) in the *iTools* ontology interface. This will invoke the display of the complete description of the chosen resource in the main viewing panel. The (expert) user can then modify any field there to update the resource meta-data. Automatic computer-based updates are facilitated (for a single resource or an entire center) by right-clicking the tree panel, and selecting “Update Resource”. These auto-updates are done by scraping existent tool-description pages (e.g., http://na-mic.org/Wiki/index.php/SDIWG:NCBC_Software_Classification_CCB_Examples). This functionality also enables users to supply a specific URL upon which the automatic update will be based. One may also update a resource by providing a local file system file (e.g., file:///path/to/thefile/). Another automated update interface for resources, or collections of resources, involves using a URL/file-system CSV (comma separated values) file. Selecting a “.csv” file (http://path/file.csv for html protocol or file:///path/to/file.csv for local file system files) will initiate the resource or collection update from an appropriate source. This is useful as frequently one can export an existing database or XLS file to .csv and then use this dump to update the *iTools* resource/center meta-data.

### *iTools* Plug-ins, Extensions, Interfaces and Database

The *iTools* design and Java-implementation include a small resource core that contains the foundational *database* and *interface* objects and methods. These provide the core functionality for future extensions and plug-ins that may improve performance and facilitate the *iTools* graphical (e.g., skins), manipulation (e.g., traversal/search) and computational (e.g., graphical workflows) utilities. Such plug-ins may be developed by the NCBCs or the outside community.

*iTools* core *interfaces* include human-user and machine-driven portals for browsing, searching, revising and comparing of the resources in the *iTools* resource database. The *human-readable interfaces* include a Java WebStart, HTML and XML browsers. The most commonly used human interface naturally is the HTML browser as it allows the fastest and most intuitive access to the *iTools* resources. The *computer interfaces* provided in the core *iTools* infrastructure include SOAP, XML and WSDL. These are selected to provide the basic means of searching or updating *iTools* because these three are currently the main protocols for platform-, OS- and language-independent ways of communication and information exchange between different computer programs. An additional advantage provided by these interfaces is that such communications are much more likely to get through firewall servers that screen out requests other than those for trusted applications.

At installation time, the core *iTools database* configuration properties allow for one of two types of Data Access Object (DAO) resource meta-data management infrastructures – file-system or hibernate. The *hibernate* option allows access to any available external database server, including MYSQL, DERBY, JDBC, and the local *file system* option provides access to a XML file. The *iTools* ANT-based build file allows seamless conversion from one type of resource meta-data storage to another at any time without interruption of service. There are four basic *iTools* database tables – *User* to store user specific information; *AllResources* and *AllProperties* to store resource meta-data and *ResourceReviews* to store reviews and user comments and rankings of *iTools* resources. The details about the *iTools* database are also online (http://cms.loni.ucla.edu/iToolsDatabase.aspx).

### *iTools* Web-crawler Plug-in

**Figure 2:**
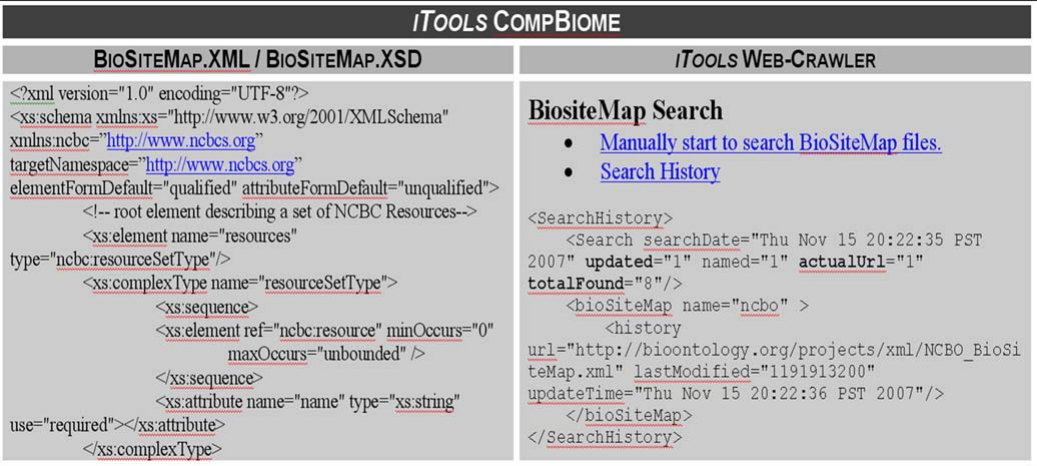
*iTools CompBiome *– the *iTools* Computational Biology Resourceome plug-in consists of a decentralized collection of BioSiteMaps (*sitemaps* of resources for biomedical computing) and a *Yahoo!Search*-based crawler for discovering new and updating existent BioSiteMaps anywhere on the web. These updates propagate automatically to *iTools*' SandBox and are later reviewed by expert users for inclusion in the *iTools* DB. The distributed nature of the NCBC CompBiome may be utilized by any tool developer, user or librarian to find, compare, integrate and expand the functionality of different resources for biomedical computing. The left and right panels illustrate the XML schema definition for the BioSiteMap.xml files and the results of a *manual* initiation of the *Yahoo!Search* using the *iTools* CompBiome plug-in, respectively. *iTools* has an automated weekly crawler initiation as well as manual triggering of the crawler.

We have prototyped an *iTools* plug-in (Computational Biology Resourceome, CompBiome) that enables decentralized resource management and updating. This plug-in is based on the *Yahoo Search! Web API* interface and blends the features of RSS feeds and sitemaps (www.sitemap.org) to facilitate distributed and efficient computational resourceome management. CompBiome inherits the simplicity and the functionality of RSS feeds to disseminate resource descriptions via an XML broadcasting model, where each site developing computational biology resources may distribute the specifications of their resources according the XSD/XML schema (www.biositemap.org/biositemaps-v04.xsd). At the same time, *iTools* uses the Yahoo! Search engine to discover new and updated computational biology resource site maps (BioSiteMaps.XML), which are parsed and their content automatically stored in the *iTools* SandBox. Expert *iTools* editors then review these SandBox entries and update the core *iTools* database with new and revised resource descriptions accordingly, **Figure 2**.

### *iTools* HT Resource Browser

**Figure 3:**
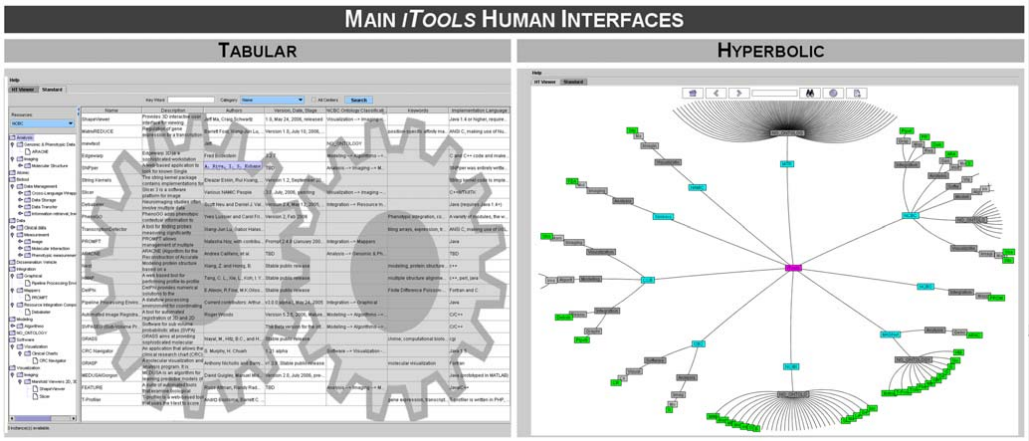
The main two displays of *iTools* resources provide tabular (left) and graph-based (right) human interfaces to the resource database (http://*iTools*.ccb.ucla.edu/). Both of these facilitate comprehensive traversal, comparison and search of resources. There are several other human and machine interfaces to the *iTools* database which are discussed in the text.

In addition to traversing, browsing and searching the *iTools* database using the NCBC ontology tree and the tabular framework, we provide an interactive hypergraph display [14] that significantly improves the visual representation of the entire collection of resources, **Figure 3**. The hierarchical visualization of graphs on non-Euclidian hyperbolic plane and subsequent mapping to the Euclidian space (2D ellipse) was introduced by [15]. The relevance of this graph embedding and visualization is rooted in the inherited features of the NCBC resource ontology (http://Bioportal.Bioontology.org): (1) There are currently hundreds of biomedical and computational resources included in *iTools* hierarchy (including resources from IATR and the 7 NCBCs). As other investigators and centers adopt the BioSiteMaps protocol, this collection is expected to rapidly grow in the 100,000's, which demands a new way of graph display and traversal; (2) The user will be interested in the relationships between different resources, represented by their ontological classification, for determining appropriate protocols for resource integration and interoperability; (3) Hyperbolic graph displays give the perception of having interactive access to all nodes at the same time, despite the fact that only a small number of nodes (for large graphs) are actually rendered on the screen. The mathematics behind these hyperbolic space transformations have been worked out much earlier, but have had limited applications in other research areas [16], [17]. The *iTools* hyperbolic viewer uses non-Euclidian geometry because in hyperbolic spaces, circle circumferences grow exponentially as functions of the distance from the (elliptical) space center. Thus, exponentially more space is available (albeit at lower resolution) as a “radial” function of the distance from the center. In a way, this is exactly the way the eye sorts complex scenes by focusing on a pivotal object or feature and the farther away another object or feature is the less information is processed by the eye, because of the lens effect of the cornea. In this case, *iTools* resource nodes may grow exponentially, as composite nodes on a tree, with increasing distance from the root (center). As **Figure 3** shows, this mapping allocates more space to the central nodes, within the middle of the hyperbolic ellipse space, and conversely nodes that are distant relative of the current central node are densely packed in the periphery (ellipse boundary). At the start, the *iTools* hyperbolic viewer presents a graph centered at the root of the hierarchy (*iTools*). Traversing, browsing and searching of the *iTools* resource hierarchy is achieved interactively via mouse actions or button clicks. There are also other agile and dexterous strategies for rendering, manipulating and traversing complex hierarchies. For example, the fractal viewer [18] and the H3 viewer [19] provide fractal self-similarity based and 3D hyperbolic structure means for displaying very large hierarchies, respectively. Either of these, or other, visualization frameworks may be integrated as *iTools* plug-ins in the future.

## Results

### Biomedical Imaging Applications

Despite the variety of scales, modalities, formats and study goals, a typical biomedical image analysis protocol consists of three parts – image preprocessing, analysis and interpretation. Each of these components may involve a sequence of steps built on available general or application/problem-specific tools. Construction and validation of such image analysis protocols is a two tier process. At the higher level, an expert outlines the pseudo-specific steps that the data needs to go through (e.g., format conversion, filtering, spectral analysis, statistical mapping and visualization). The lower-level implementation of the image analysis protocol demands the exploration of specific computational tools and resources that implement the required sequence of data processing steps. *iTools* facilitates this (lower-level) design process by providing comprehensive interfaces to a large, expandable and descriptive imaging resource meta-data archive. Another direct application of the *iTools* infrastructure is providing the investigator with the ability to construct meta-algorithms and meta-tools for computational biology by finding and linking existent resources. Development of meta-algorithms holds a lot of promises since such meta-resources typically have various optimality properties [20].

### Biomedical Informatics

The field of biomedical informatics is rapidly approaching the era of personalized, evidence-based and multispectral medicine. Such shift will inevitably increase the complexity of native and processed patient data stored in various Electronic Health Record formats. Both, the observed and the processed data will increase in volume and complexity. The flexible and extensible *iTools* infrastructure allows for management of disparate computational biology resources, which includes databases. A direct application of the *iTools* framework is the ability of a researcher to store the provenance of the data that was processed through a stream of computational tools. This *iTools* feature is not implemented yet, however, this plug-in is currently being designed. This functionality will allow finding computational tools (within *iTools*), filtering data accordingly (outside *iTools*) and storing the snapshot of the state of the tools used in this process. This data history may then be used to apply the same analysis protocol to new data in the future. The ontological classification provided by *iTools* may be used to facilitate revisions, updates and expansions of such data provenance.

### Graphical Workflow Tool Integration

**Figure 4:**
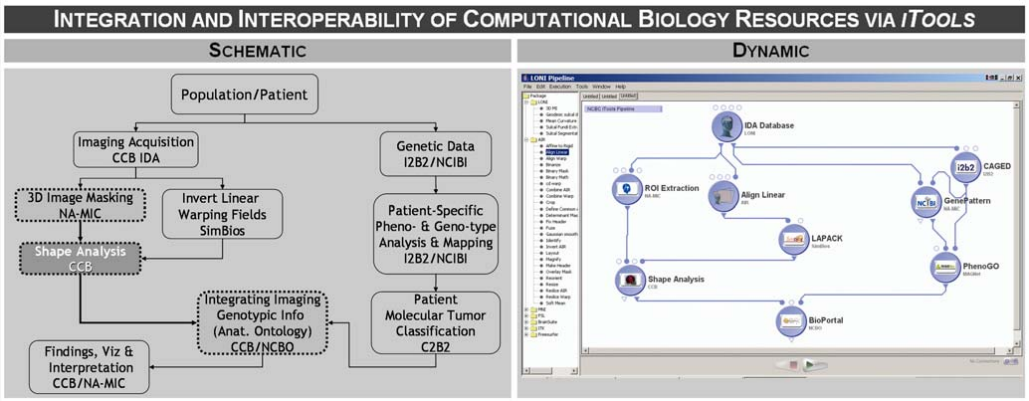
A schematic and dynamic integration of *iTools* resources demonstrating interoperability of multi-disciplinary tools via graphical workflow environments. The three nodes with dash-boundaries on the *left* demonstrate schematically the integration of some computational biology tools. The graphical workflow on the *right* depicts the practical means of using *iTools* meta-data to construct module descriptions and generate multidisciplinary and heterogeneous data analysis protocols.

One of the critical long term-goals of the computational biology and bioinformatics community is to establish an agile infrastructure that allows integration and interoperation of disparate, heterogeneous and multidisciplinary tools. Two fundamental components are required to achieve progress in this direction – a database of resources (e.g., *iTools*) and an infrastructure for mediating resource communications, including data transfers. As part of the optional resource meta-descriptors, *iTools* enables resource curators and librarians to provide a complete description of the type, format and specifications of any input or output data for each resource archived in *iTools*. For example, suppose we have a tool that segments the mask of an organ and provides this result as input to a second independent tool. The latter may be image-analysis software that takes as input the binary mask image generated by the first tool and computes a signature vector of shape characteristics of the organ. Then, a third resource may integrate the shape description vector produced in step two with clinical, demographic or genetic patient data to allow for such multispectral analysis of the patient and organ of interest. The three nodes marked with dash-boundaries in the left panel of **Figure 4** provide a schematic representation of this pseudo-experiment. The graphical workflow on the right panel in **Figure 4** demonstrates a practical dynamic representation of this resource integration example. The *iTools* infrastructure greatly facilitates this type of multi-resource interactions and interoperability.

**Figure 5:**
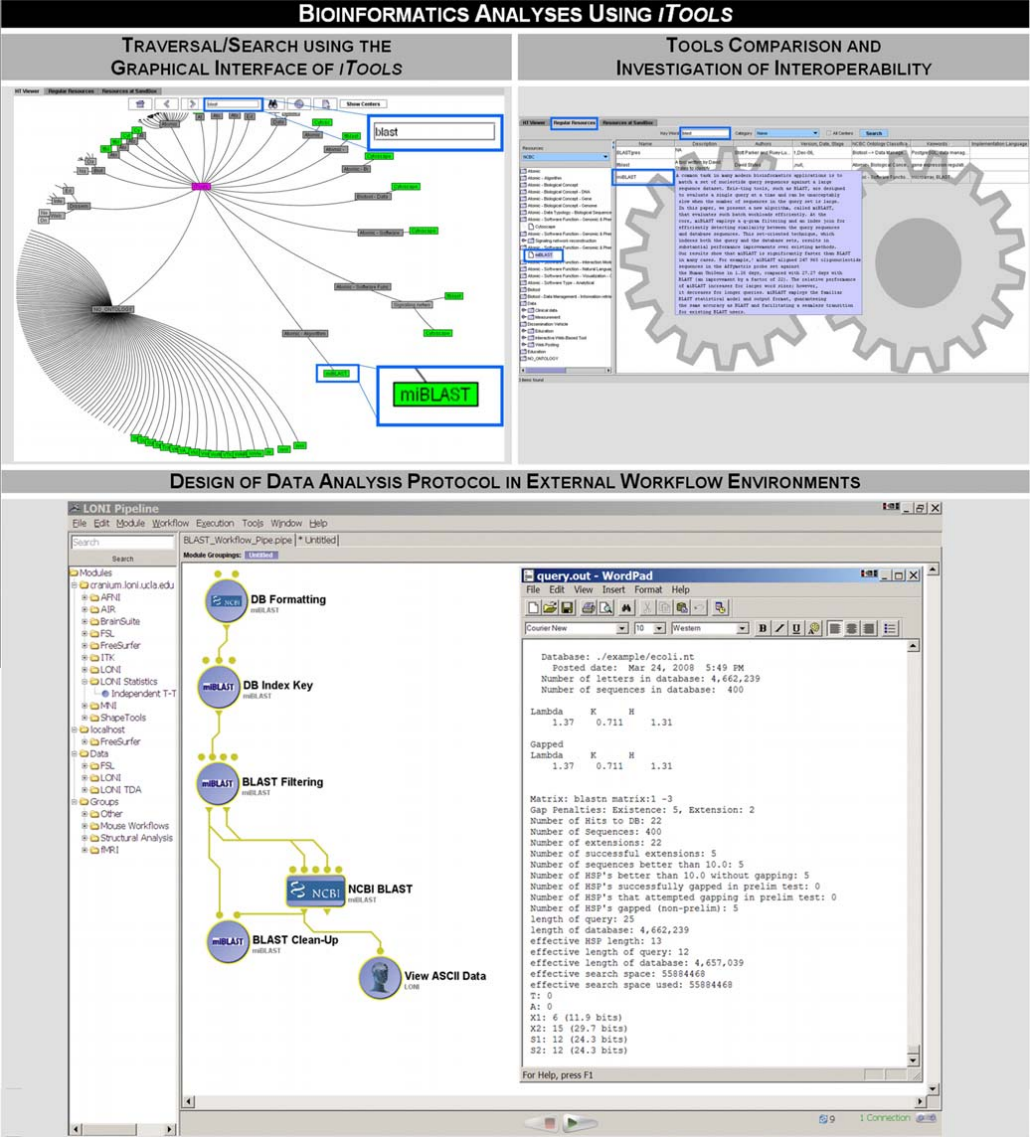
This figure illustrates the utilization of *iTools* for search, comparison and integration of bioinformatics tools. In this example, we demonstrate the use of the Basic Local Alignment Search Tool (BLAST) for comparing gene and protein sequences against other nucleic sequences available in various public databases. The *top row* shows *iTools* traversal and search (keyword = blast) using the hyperbolic graphical interface, and tools comparison and investigation of interoperability using the tabular resource view panel. The *bottom row* shows the design of a simple BLAST analysis workflow using one specific graphical workflow environment (LONI Pipeline). This BLAST analysis protocol depicts the NCBI DB formatting, index generation and filtering using *miBLAST*, sequence alignment and result textual visualization.

**Figure 5** demonstrates another *iTools* example using bioinformatics resources. We use the commonly used Basic Local Alignment Search Tool (BLAST, [21], [22]) which finds regions of similarity between biological sequences (typically data and references available in various public databases). The top row in **Figure 5** shows the *iTools* graphical traversal and keyword search (key = blast) using the graphical interface and the tabular resource view panel. The bottom row shows the design of a simple BLAST analysis workflow using one specific graphical workflow environment (LONI Pipeline, [23]). This BLAST analysis protocol depicts the NCBI DB formatting, followed by index generation and filtering using *miBLAST* [22], NCBI BLAST sequence alignment and result textual visualization. This workflow is available for testing in the collection of LONI Pipeline's bioinformatics resources (http://Pipeline.loni.ucla.edu, [23]). It is important to clarify that *iTools* does not include a tool-integration environment, nor does it include the binary executable components of the corresponding computational biology resources. *iTools* provide the infrastructure for a common resource description and dissemination, manages the meta-data about these resources, and enables the investigation of resource interoperability. Using the actual resources may require web-service accounts, downloading and installation, conversion and integration of resources which need to be accomplished outside of *iTools*.

**Figure 6:**
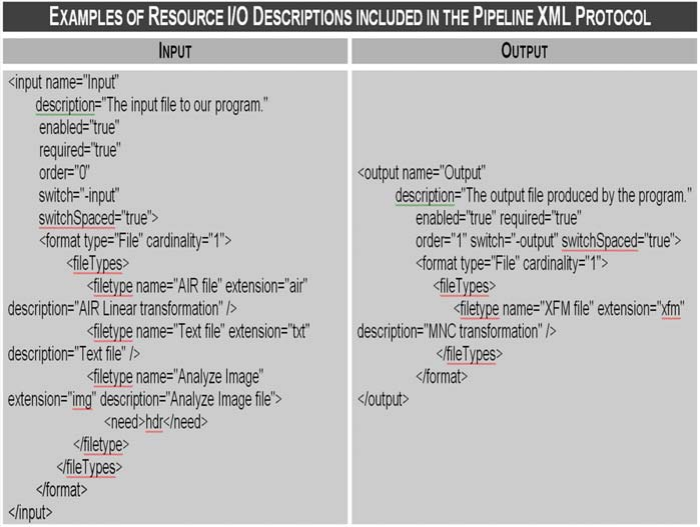
Examples of the *input* and *output* XML descriptions in the Pipeline, an integrated graphical workflow environment that mediates inter-resource communications. If resources described in *iTools* include such data I/O descriptions, external interoperability environments (like the Pipeline) will be able to automatically enable construction and validation of inter-resource computational workflows.

Only the meta-descriptions of resource *inputs* and *outputs* (I/O) are stored in *iTools*. These I/O (data, control and parameter) fields are interpreted by the mediating resource-integrating environment. For instance, **Figure 6** illustrates the XML protocol adopted by the LONI Pipeline (http://cms.loni.ucla.edu/pipeline/default.aspxid3234termsXML) to enable efficient, robust, unique and extendible description of resource I/O parameters.

To present a complete hands-on illustration of the utilization of *iTools*, using real-data, we present a neuroimaging case study. The research aim of this study is to construct a new *elderly female Alzheimer's Disease MRI brain atlas* that enables volume-based morphometric analyses (e.g., white matter, gray matter and cerebro-spinal fluid volume measures across individuals). To achieve this goal, we use *iTools* to find appropriate data (DB search), applicable software tools and enable the integration of these resources within a well-defined analysis protocol. This protocol is designed, validated and disseminated as an XML graphical workflow via the Pipeline or any other tool integration environment. The complete details of this case study are available online at: http://cms.loni.ucla.edu/iTools/integration.aspx.

## Discussion

### Challenges, approach and results

The computational biology field greatly increased the demand for, and supply of, new decentralized, portable, scalable, and lightweight resources for data localization, management, processing, analysis and visualization. This rapid increase of the quantity and quality of newly available tools for biomedical research is not fully utilized in practice because of the lack of universally accepted standards and a common resource management infrastructure. For example, finding, comparing, classifying, ranking and exploring the wide spectrum of tools developed by multiple disciplines prove challenging for many investigators. This leads to suboptimal (sometimes improper) practical engagement of powerful new computational algorithms and tools.

To facilitate this interface between the computational biology research needs and the available resources, we propose a new extensible, lightweight and portable framework (*iTools*) for resource management, evaluation and mediation. *iTools* is developed as an open-source, open-content and open-use resource, which provides a secure community-based infrastructure for resource management, communication and integration of resources developed by investigators for a wide gamma of applications. We based our design and development on the broad research, computational and translational science expertise available at the seven National Centers for Biomedical Computing. The current *iTools* resource ontology and archive of resources provides a general framework for discovering and utilizing various computational biology resources. Furthermore, the *iTools'* design allows both resource meta-data and infrastructure expansions, which will appeal to many investigators.

### Technical details

The *iTools* framework is available in three forms. The most popular one is the *iTools* server, which provides human and computer interface to the current *iTools* resource descriptions. Access to the *iTools* server is provided via www.NCBCs.org and http://iTools.ccb.ucla.edu. The other two distribution forms of *iTools* include source (Java/HTML/XML) and binary (JAR) downloads. These are provided to facilitate the development of *iTools* plug-in as well as the utilization of the *iTools* framework in other areas of science, technology and education. The current version of *iTools*, as a meta-resource for computational biology resources, includes hundreds of different software tools, data archives and web-services, some provided by the NCBC community and some developed by other initiatives. The existent *iTools* resources incorporate tools, databases and services for modeling, analysis and visualization of biological images, genotypic sequences and clinical datasets. Any (registered) user, investigator or resource developer may submit a resource for inclusion in *iTools*. All submissions are carefully reviewed by 3 expert reviewers before their migration to the publicly viewable component of the *iTools* meta-database. The *iTools usage guidelines* (http://iTools.ccb.ucla.edu) provide details on how to extend, utilize and manage updates and revisions of *iTools* resources. The *iTools* framework is flexible, agile and extensible in two directions. First the core and plug-in computational component of *iTools* may be readily widened to include new and revise existent *iTools* functionality. Committing these changes to the *iTools* code requires expert editor approval. The second type of *iTools* expansion involves its maturation and the accrual of new resources or types of meta-resources (e.g., IATR, [24]). The growth and progress in both of these directions is entirely dependent on the community involvement and contributions.

### *iTools* Validation

To date, we have done limited testing of the *iTools* infrastructure by including diverse types of NCBC resources, validating the human and machine interfaces and exploring the management of the *iTools* database infrastructure. As we have deployed on the web a stable version of the software, we have opened this framework for contribution, assessment and feedback by the larger computational biology community. In the next 18 months, we will collect, analyze and report statistics on *iTools* usage, query, update and expansion logs. Such activity measures will provide valuable information about the scope of adoption, usability and extendibility of the newly proposed Resource Ontology (http://Bioportal.Bioontology.org), XML resource description format (www.biositemap.org/biositemaps-v04.xsd) and the Computational Biology Resourceome (CompBiome).
